# Glycogen Synthase Kinase-3β Is Associated with the Prognosis of Hepatocellular Carcinoma and May Mediate the Influence of Type 2 Diabetes Mellitus on Hepatocellular Carcinoma

**DOI:** 10.1371/journal.pone.0105624

**Published:** 2014-08-26

**Authors:** Guoliang Qiao, Yuan Le, Jun Li, Lianghuan Wang, Feng Shen

**Affiliations:** 1 Department of Hepatic Surgery, Eastern Hepatobiliary Surgery Hospital, Second Military Medical University, Shanghai, China; 2 Department of Biochemistry and Molecular Biology, Second Military Medical University, Shanghai, China; The University of Hong Kong, China

## Abstract

**Background:**

Although many studies have shown glycogen synthase kinase-3β(GSK-3β) was associated with type 2 diabetes mellitus(T2DM) and implicated with a wide range of cancers, the role of GSK-3β in hepatocellular carcinoma(HCC) and the correlation among GSK-3β, T2DM and HCC remains unclear. Our objectives were to identify the effect of p-Ser9-GSK-3β on the prognosis of patients with HCC and to learn more about the interaction among T2DM, GSK-3β and the prognosis of HCC.

**Methods:**

Firstly we used reverse transcriptase-PCR(RT-PCR) and western blotting to determine the expression levels of GSK-3β and p-Ser9-GSK-3β in human HCC samples. We then used immunohistochemical staining to evaluate the expression pattern of p-Ser9-GSK-3β in 178 patients with HCC after curative partial hepatectomy. Finally we statistically analyzed the association of p-Ser9-GSK-3β and T2DM with the prognosis of patients with HCC.

**Results:**

P-Ser9-GSK-3β was over-expressed in tumor tissues compared with their normal counterparts. Correlation and regression analysis indicated that the over-expression of p-Ser9-GSK-3β was significantly associated with T2DM, and the correlation coefficient was 0.259 (P = 0.001). Multivariate analysis showed that the over-expression of p-Ser9-GSK-3β(P<0.001) and T2DM(P = 0.008) were independently associated with poor prognosis of HCC, respectively. Further analysis demonstrated that these two variables are closely related with each other.

**Conclusion:**

The over-expression of p-Ser9-GSK-3β and T2DM are strongly correlated with worse surgical outcome of HCC. P-Ser9-GSK-3β may play a significant role in mediating the influence of T2DM on the prognosis of HCC.

## Introduction

Hepatocellular carcinoma(HCC) is the fifth most common malignancy worldwide and the second most frequent cause of cancer-related mortality [1]. Partial hepatectomy remains the standard curative treatment, with a 5-year survival rate of 50%–70% for early HCC [2], [3]. Accurately identifying the prognostic factors is important to facilitate screening of high risk patients and make decisions on the adjuvant therapy.

Glycogen synthase kinase 3 (GSK3), a constitutive multi-functional serine threonine kinase, was involved in diversely physiological pathways ranging from insulin signaling pathways which associated with the disease of type 2 diabetes mellitus (T2DM) and Wnt signaling pathways which related to oncogenesis [4]–[7]. GSK-3β (47 kDa), an isoform of GSK-3, could be inactivated by insulin through phosphorylation of an N-terminal domain serine residue(Ser9 of GSK-3β) [8]. Phosphorylation of Ser9-GSK-3β (p-Ser9-GSK-3β) was associated with the activation of Wnt signaling which could prevent phosphorylation and lead to the stabilization of β-catenin. This mechanism had been reported in a wide range of cancers [9]. However, few researchers reported the significance of GSK-3β or p-Ser9-GSK-3β in the prognostic evaluation of patients with HCC [10].

Currently, epidemiology studies revealed that diabetes mellitus(DM) increased the incidence of HCC [11], [12]. Although it remained controversial about the influence of DM on the prognosis of patients with HCC after partial hepatectomy [13], [14], accumulating evidence showed that DM was an independent prognostic indicator for patients with HCC after curative resection [15], [16]. The purpose of the present study is to identify if the expression level of p-Ser9-GSK-3β is an effective prognostic factor for HCC patients and learn more about the interaction among the T2DM, expression of p-Ser9-GSK-3β and the prognosis of HCC.

## Materials and Methods

### 1. Patients and human HCC specimen

A total of 238 patients with HCC undergoing partial hepatectomy by two independent surgical teams at Eastern Hepatobiliary Surgery Hospital from July 1^st^, 2003 to May 15^th^, 2005 were enrolled in this study. The preoperative clinical diagnosis of HCC met the diagnostic criteria of the American Association for the Study of Liver Diseases(AASLD) [17]. Of these surgical HCC specimens, 60 samples which were collected between July 1^st^ and December 15^th^, 2003, were subjected to RNA and protein extraction that could be done for RT-PCR and western blot analysis. 178 patients collected between December 16^th^, 2003 and May 15^th^, 2005 met the following inclusion criteria and then underwent immunohistochemical staining (IHC) and prognostic analyses: had WHO performance status 0-1; did not receive any preoperative anti-cancer therapy; did not have any distant metastasis, image visible ascites, or severe esophageal varices; had the preoperative Child-Pugh class A; received curative hepatectomy; had pathologically proven hepatocellular carcinoma. Patients were excluded from this study if they had a history of other solid tumors, or died from severe postoperative complications. The clinical characteristics of the 178 patients were listed in Table 1.

**Table 1:** Correlation between p-Ser9-GSK-3β expression and clinicopathologic features.

Variables		p-Ser9-GSK-3β expression		
		Low(n = 89)	High(n = 89)	*P*
Sex	female	12	14	0.671
	male	77	75	
Age	median	50	49	0.613
	range	15–72	32–74	
AFP level(µg/L)	≤400	53	54	0.878
	>400	36	35	
HBsAg	positive	79	78	0.816
	negative	10	11	
HBeAg	positive	20	13	0.177
	negative	69	76	
Liver cirrhosis	yes	69	75	0.253
	no	20	14	
Edmondson-Steiner grade	I-II	17	20	0.579
	III- IV	72	69	
Diameter(cm)	≤5	52	51	0.879
	>5	37	38	
Encapsulation	complete	32	21	0.071
	no	57	68	
MVI	yes	29	34	0.433
	no	60	55	
Tumor number	single	76	70	0.242
	multiple	13	19	
TBL(umol/l)	median	15.4	16	0.603
	range	4.2–56.7	4.6–64.4	
Alb(g/dl)	median	38.4	40	0.092
	range	24.5–51.4	23.6–53.8	
ALT(U/L)	median	54.9	55.5	0.887
	range	10,6–235.7	8.3–240.3	
WBC	median	5.2	5.1	0.611
	range	2.5–13.7	3.1–14.8	
RBC	median	4.4	4.3	0.348
	range	3.2–5.8	2.8–5.4	
PLT(*10^9^/L)	median	124	122	0.783
	range	22–359	17–367	
INR	median	1.05	1.04	0.308
	range	0.87–1.26	0.89–1.23	
CR	median	70	68	0.454
	range	40–136	38–139	
MELD score	median	7.6	7.5	0.564
	range	6.0–12	6.0–11	
T2DM	yes	6	23	0.001
	no	83	66	

Abbreviations: AFP, alpha-fetoprotein; HBsAg, hepatitis B surface antigen; HBeAg, Hepatitis E antigen; MVI, microvascular invasion; TBIL total bilirubin; ALB, albumin; ALT, alanine; WBC, white blood cell; RBC, red blood cell; PLT, platelet count; INR, international normalized ratio; CR, creatinine; MELD, model for end-stage liver disease; T2DM, type 2 diabetes mellitus.

The definition of curative hepatectomy was described as our previous reports [18]. The histological grade of tumor differentiation was assigned by the Edmondson Steiner grading system [19]. This study was approved by the Institutional Review Board of Eastern Hepatobiliary Surgery Hospital. All patients gave written informed consent to participate. The parents wrote the informed consent on behalf of the patients whose age<18. The ethics committees approved this consent procedure. The data did not contain any information that could identify the patients.

### 2. Immunohistochemistry and evaluation of immunostaining

Immunohistochemical staining was performed with the Dako Envision Plus System (Dako, Carpinteria, CA) according to the manufacturer's instructions. The primary antibodies were anti- GSK-3β and anti-p-Ser9-GSK-3β (Cell Signaling Technology Inc.,Beverly, MA, 1∶50). The tissues were evaluated as positive for GSK-3β and p-Ser9-GSK-3β staining when there were more than 10% of tumor cells demonstrating cytoplasmic and/or nucleus immunoreaction deposits. The sections were scored with a four-tier scale: 0 = negative (0–10%), 1 = weak signal (10–20%), 2 = intermediate signal (20–50%) and 3 = strong signal (> 50%). 0 and 1 were defined as low exprression, while 2 and 3 were defined as over-expression. All sections were scored independently by two observers who did not have any prior knowledge of the clinic-pathologic data. The concordance between scores from different sections of the same tumor was greater than 90%. All discrepancies in scoring were reviewed and a consensus was reached.

### 3. Western blotting analysis

Fresh surgical specimens were snap frozen in liquid nitrogen and stored in deep freezer. The normal and the tumor tissues were lysed in T-PER Tissue Protein Extraction Reagent(Pierce, Rockford, IL) containing proteinase inhibitors(CalBiochem, San Diego, CA). The extractions were collected and centrifuged at 12,000×g for 5 min. The protein concentrations were determined using the BCA Protein Assay (Pierce) according to the manufacturer's instructions. The following antibodies were used: anti-GSK-3β and anti-p-Ser9-GSK-3β (Cell Signaling Technology Inc., Beverly, MA), we also used β -actin as a loading control.

### 4. RNA extraction and real-time reverse transcriptase-PCR

Total RNA of tissue samples were isolated using TRIzol (Life Technologies, Inc., Rockville, MD) according to the manufacturer's instructions. cDNA was generated from 1 ug of each RNA sample and a reverse transcribed using a transcription kit (Takara, Kyoto, Japan). Real-time quantitative reverse transcriptase-PCR (RT-PCR) was done in the 7300 Real Time PCR System (Applied Biosystems) to detect GSK-3β gene expression in paired liver samples from 30 HCC patients. The primers were as follows: GSK-3β, forward: 5′-AACACCAACAAGGGAGCAAA-3′; reverse: 5′-GAGCGTGAGGAGGGATAAGG-3′, 326bp. β-actin, forward: 5′-ACCATGGATGATGATATCGC-3′ and reverse: 5′-ACATGGCTGGGGTGTTGAAG-3′, 386bp. β-actin served as an endogenous control. The expression of the target messenger RNA(mRNA) was quantified relative to that of the β-actin mRNA. A ratio of relative GSK-3β mRNA levels in tumor samples/nontumorous samples of 0.5-fold was defined as under-expression of the gene, whereas a ratio of 2.0-fold was defined as over-expression.

### 5. Diagnosis of Type 2 diabetes

Diagnosis of diabetes mellitus was based on the 1999 World Health Organization criteria. Patients who were found to have a fasting blood sugar level between 5.6 and 6.1 mmol/L were defined as having impaired fasting glucose and were referred for confirmation of their diabetes status. Those with a fasting blood sugar level of at least 7.0 mmol/L or those diagnosed with Type 2 diabetes before entering the hospital were defined as having Type 2 diabetes and were referred for further diabetic care. Patients with prior diagnosis of Type 1 diabetes were excluded from the study.

### 6. Follow-up

The follow-up ended on May 2012 and the median follow-up duration was 48.2 months (3.8–114.5 months). Contrast-enhanced CT scan or MRI of the abdomen was carried out once every 3 months in the first two years after surgery, and then once every 6 months thereafter. The diagnostic criteria for HCC recurrence was the same as used for the initial diagnosis.

### 7. Statistical analysis

Overall Survival (OS) and Time To Recurrence (TTR) were used as primary endpoints. OS was defined as the interval between hepatectomy and death or the date of last follow-up. TTR was calculated from surgery to the date when recurrence was diagnosed. All statistical calculations were carried out using the SPSS.16.0 software. The χ2 test or Fisher's exact test were used to compare qualitative variables, while continuous variables were compared using Student's t-test or Mann-Whitney test for variables with an abnormal distribution. Receiver operating characteristic curve analysis was used to determine the optimal cut-offs of continuous variables. Survival curves were calculated by the Kaplan-Meier method and compared using the log-rank test. The Cox proportional hazards model was used to determine the independent factors for tumor recurrence and patient's survival based on the variables selected by univariate analysis. Differences were considered statically significant at p< 0.05.

## Results

### 1. Over-expression of GSK-3β and p-Ser9-GSK-3β in HCC samples

**Figure 1:**
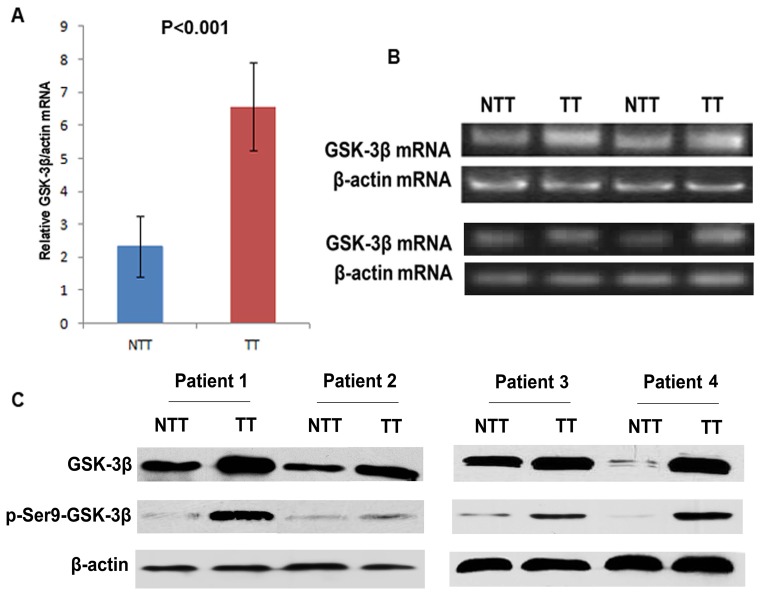
Expression of GSK-3β and p-Ser9-GSK-3β in HCC. (A) Relative expression level of GSK-3β mRNA in 60 tumor tissues(TT) and paired non-tumorous tissues(NTT) by RT-PCR (P<0.001). (B) RT-PCR analysis of GSK-3β mRNA expression. (C) Expressions of p-Ser9-GSK-3β were analyzed by Western Blotting in HCC tumor tissues(TT) and paired non-tumorous tissues(NTT).

**Figure 2:**
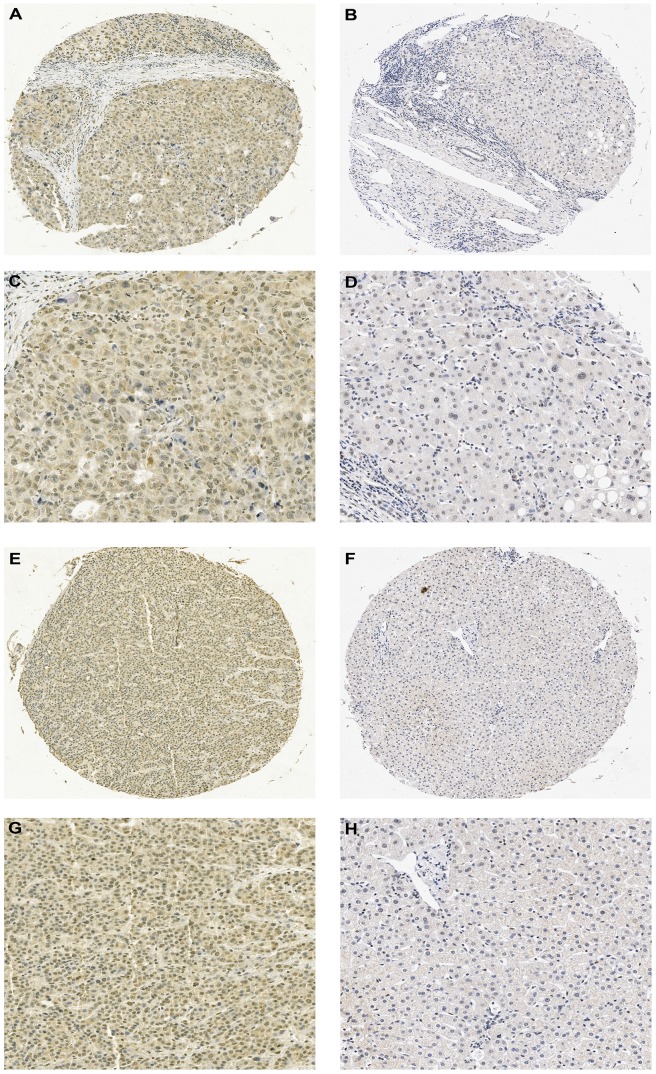
Immunohistochemical analysis of p-Ser9-GSK-3β expressions in HCCs and the adjacent nontumorous liver tissues. (A-D) Immunohistochemical staining of a paired HCC and nontumoral tissue. HCC cells were strongly positive for p-Ser9-GSK-3β expression in the cytoplasm (A,C). Normal hepatocytes in the nontumoral tissue showed no detectable p-Ser9-GSK-3β expression(B,D). (E-H) Immunohistochemical staining of another paired HCC and nontumoral tissue. HCC cells were strongly positive for p-Ser9-GSK-3β expression in the cytoplasm(E,G). Normal hepatocytes in the nontumoral tissue showed no detectable p-Ser9-GSK-3β expression(F,H). Original magnifications: magnification*50 (A,B,E,F); magnification*100 (C,D,G,H).

We firstly assayed and compared GSK-3β mRNA levels in 60 TTs(tumor tissues) and their matched NTTs(nontumorous tissues) by the way of RT-PCR assay (Fig. 1A, B), in which 50 of 60 (83.3%)specimens showed GSK-3β mRNA over-expression in HCC(tumor/nontumorous ratio>2.0). . We found increased protein expression level of total GSK-3β and p-Ser9-GSK-3β in 46 of 60(76.7%)tumor tissues compared with their normal counterparts by western blotting (Fig. 1C). We then performed immunostaining in the 178 paired HCC samples and found that 89(50.0%)patients were identified as p-Ser9-GSK-3β over-expression (Fig. 2A-H)

### 2. Association of p-Ser9-GSK-3β expression with T2DM

We examined the association between the expression level of p-Ser9-GSK-3β in the tumor tissues with T2DM and other clinic-pathological characteristics of patients (Table 1). Correlation and regression analysis indicated that the over-expression of p-Ser9-GSK-3β was significantly associated with T2DM, and the correlation coefficient was 0.259 (P = 0.001).

### 3. Association of p-Ser9-GSK-3β expression and T2DM with prognosis of HCC patients

Patients with over-expression of p-Ser9-GSK-3β had higher recurrence rates (1-,3-,and 5-year recurrence rates: 49.4%, 70.0% and 84.7% vs. 24.9%, 43.1%, and 63.9%, P<0.001), and lower overall survival rates (1-,3-,and 5-year survival rates: 73.6%, 44.8% and 34.9% vs. 88.5%, 72.8% and 61.5%, P<0.001) than those with low expression.

T2DM was negatively correlated with 1-,3-, and 5-year survival rates (64.6%, 39.7% and 29% for T2DM vs 84.2%, 62.2% and 51.7% for No T2DM; P < 0.001). The 1-,3-, and 5-year cumulative recurrence rates in patients with T2DM were significantly higher than those in patients with No T2DM(58.6%, 78.9% and 91.6% vs 33.1%, 52.4% and 71.2%; P<0.001).

**Figure 3:**
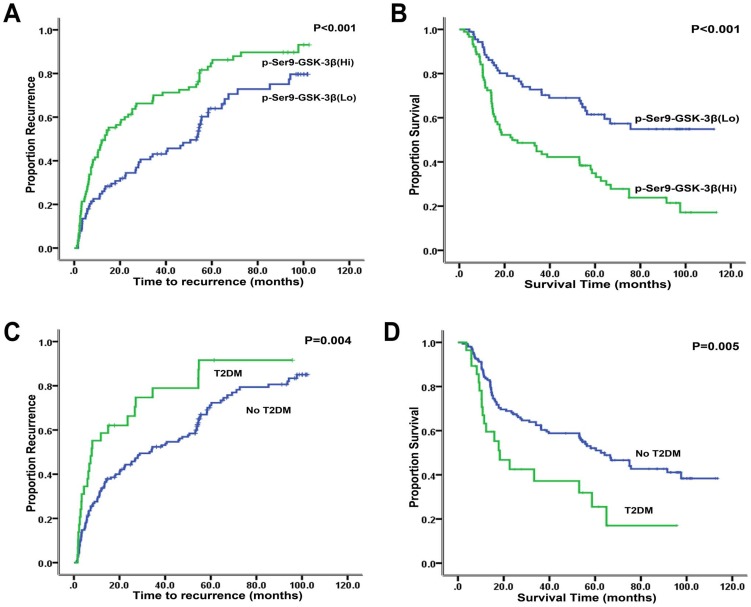
Kaplan-Meier analysis of TTR and OS of patients with HCC. (A,B) Kaplan-Meier curves of the correlation between p-Ser9-GSK-3β expression level and (A) recurrence or (B) overall survival of HCC patients; (C,D) Kaplan-Meier curves of the correlation between T2DM and (A) recurrence or (B) overall survival of HCC patients.

Based on Kaplan-Meier survival analysis, the comparisons of p-Ser9-GSK-3β expression levels(high or low) in the 178 patients with HCC were shown in the Fig 3A, B. The comparisons of patients with no T2DM versus T2DM were shown in the Fig 3C, D.

Univariate analysis of 23 survival-related clinic-pathological variables revealed that serum AFP level(P  = 0.024), tumor size(P = 0.003), tumor number(P = 0.022), liver cirrhosis(P = 0.038), microvascular invasion(P = 0.011), p-Ser9-GSK-3β expression level(P<0.001), and T2DM(P = 0.006) were statistically correlated with OS of patients with HCC (Table S1).

These significant variables were further subjected to multivariate Cox proportional hazards model, which shown that tumor size(P = 0.008), tumor number(P = 0.032), microvascular invasion(P = 0.019), p-Ser9-GSK-3β expression level(P<0.001) were independent risk factors that could affect the survival of patients with HCC(table 2). We speculated that some interaction or colinearity existed between the variables of p-Ser9-GSK-3β and T2DM. Thus, we further conducted multivariate Cox proportional hazards analysis excluding the variables of p-Ser9-GSK-3β or T2DM respectively (table 2). The results also demonstrated that these two variables were closely related with each other.

**Table 2:** Multivariate analysis for overall survival.

Variable	Overall Survival			Overall Survival(exclude T2DM)			Overall Survival(exclude pG)		
	HR	95%CI	P value	HR	95%CI	P value	HR	95%CI	P value
Tumor Size: >5cm/≤5cm	1.742	1.155–2.626	0.008	1.865	1.247–3.032	0.006	1.675	1.192–2.458	0.010
Microvascular Invasion: Yes/No	1.631	1.083–2.457	0.019	1.564	1.102–2.316	0.024	1.725	1.267–2.687	0.013
Tumor Number: Multiple/Solitary	1.823	1.256–3.582	0.017	1.697	1.378–3.214	0.015	1.587	1.145–2.453	0.020
p-Ser9-GSK-3β Expression: High/Low	2.292	1.320–3.978	0.003	2.439	1.577–3.772	0.001	_	_	_
T2DM: Yes/No	1.193	0.708–2.010	0.507	_	_	_	1.815	1.158–3.224	0.008

Abbreviations: pG, p-Ser9-GSK-3β; T2DM, type 2 diabetes mellitus.

### 4. Stratified analysis of the impact of p-Ser9-GSK-3β expression and T2DM on the prognosis of HCC patients

In the subgroup of tumor size≤5cm, p-Ser9-GSK-3β over-expressing patients had higher recurrence rates(1-,3-,and 5-year recurrence rates: 37.3%, 59.9% and 81.6% vs. 15.4%, 29.9%, and 54.6%, P = 0.002), and lower survival rates(1-,3-,and 5-year survival rates: 82%, 58.1% and 43.9% vs. 94.2%, 83.9% and 72.4%, P<0.001) than those of under-expressing patients.

**Figure 4:**
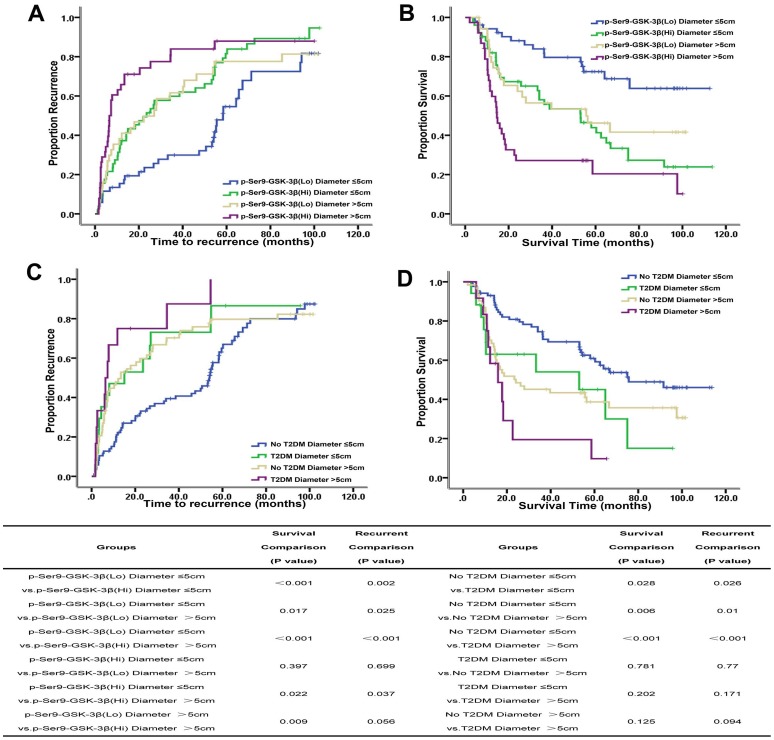
Kaplan-Meier analysis of TTR and OS stratified by tumor diameter of HCC patients. (A,B) Kaplan-Meier curves of the correlation between p-Ser9-GSK-3β expression level, tumor diameter divided by 5cm and (A) recurrence or (B) overall survival of HCC patients; (C,D) Kaplan-Meier curves of the correlation between T2DM, tumor diameter divided by 5cm and (C) recurrence or (D) overall survival of HCC patients.

Similar results were obtained in the tumor size>5 cm subgroup: the 1-,3-,and 5-year recurrence rates were 65.8%, 83.9% and 87.9% vs. 38.3%, 61.6%, and 77.6% in over-expressing and under-expressing patients, respectively, and the 1-,3-,and 5-year survival rates: 62.5%, 27.2% and 20.4% vs. 80%, 56.5% and 46.2%, respectively (P = 0.056, P = 0.009). (Fig 4A,B).

In the subgroup of tumor size≤5cm, patients with T2DM had higher recurrence rates (1-,3-,and 5-year recurrence rates: 47.1%, 73.1% and 86.6% vs. 22.2%, 39.4%, and 65%, P = 0.026), and lower survival rates(1-,3-,and 5-year survival rates: 63%, 54% and 45% vs. 93%, 74.5% and 60.9%, P = 0.028) than those of patients without T2DM.

In the tumor size>5 cm subgroup: the 1-,3-,and 5-year recurrence rates were 75%, 87.5% and 91.7% vs. 48%, 70.3%, and 79.7% in patients with and without T2DM, respectively, and 1-,3-,and 5-year survival rates: 66.7%, 19.4% and 9.7% vs. 71.9%, 45.1% and 38.7%, respectively (P = 0.094, P = 0.125) (Fig 4C,D). There were no significant differences in TTR and OS in the subgroup of tumor size>5cm, due to the limitation of the amount of patients with T2DM.

We then subdivided HCC patients with T2DM, we found that In the subgroup of without T2DM, p-Ser9-GSK-3β over-expressing patients had higher recurrence rates(1-,3-,and 5-year recurrence rates: 45.5%, 64.8% and 83.3% vs. 23.0%, 42.4%, and 57.1%, P = 0.004), and lower survival rates(1-,3-,and 5-year survival rates: 78.4%, 49.3% and 24.4% vs. 87.6%, 72.4% and 60.7%, P<0.001) than those of under-expressing patients. There were no significant differences in TTR(p = 0.308) and OS(p = 0.057) in the subgroup of with T2DM (Figure S1).

## Discussion

In the present study, we showed that the total GSK-3β and the p-Ser9-GSK-3β were increased at both mRNA and protein levels in HCC specimens. The over-expression of p-Ser9-GSK-3β was associated with the poor prognosis of patients with HCC after curative resection. We also found that T2DM was related with the poor prognosis of patients with HCC, and the expression level of p-Ser9-GSK-3β was significantly related with T2DM. We speculated that the regulation of GSK-3β might play a significant role in mediating the impact of T2DM on HCC prognosis. To the best of our knowledge, no previous study reported the impact of p-Ser9-GSK-3β expression on the prognosis of HCC and the relationship between the expression of p-Ser9-GSK-3β and T2DM.

GSK-3β was a crucial signaling molecule involved in glycogen synthesis, gene expression, cell migration, cell cycle, cellular architectural pathways and oncogenesis [20], [21]. It was a downstream target of insulin and insulin-like growth factor(IGF) receptor-dependent pathways which could result in GSK-3β phosphorylation on the serine 9 residue(p-Ser9-GSK-3β), and subsequent phosphorylation of insulin receptor substrate-1(IRS1) [22], [23]. Insulin receptor substrate (IRS) proteins were major docking proteins that mediated insulin/insulin-like growth factor 1 (IGF1) signaling in various insulin-sensitive tissues [24]. Therefore, dysregulation of GSK-3β could result in attenuating insulin signaling, which was thought to induce insulin resistance. Insulin resistance was a major pathophysiological problem underlying the initiation and progression of type II diabetes mellitus (T2DM) [25]. Moreover, Activation of Wnt signaling could lead to GSK-3β inactivation and prevented phosphorylation of β-catenin protein in the cytosol. The increased levels of β-catenin transported to the nuclear, and interacted with transcription factors LEF/TCF (lymphoid enhancer factor/T cell factor) to activate the expression of target genes like c-myc and cyclin D1, and lead to cell proliferation [21], [26]. This process was implicated with a wide range of cancers. With respect to HCC, apart from the Wnt-β-catenin pathway, insulin and insulin-like growth factor(IGF) pathways were also implicated with the development and/or progression of HCC [27]-[29]. GSK-3β was involved in these pathways and recent findings indicated that hyperphosphorylation of Ser9-GSK-3β was a hallmark of hepatocarcinogenesis. And also, GSK-3β was showed as a potential therapeutic target in various cancer involved in pancreatic, colon, renal, prostate, thyroid, ovarian cancer and leukemia [30]-[37].

Increasing evidence showed that DM was an independent risk factor for the prognosis of patients with HCC [15], [38]. We focused on T2DM because type 1 and type 2 DM might have different impacts on HCC prognosis and their pathophysiology mechanisms were different. In this study, our findings showed that T2DM was associated with poor survival of patients with HCC. In the subgroup analysis, T2DM was a significant independent risk factor in early HCC (tumor size< or  = 5cm, P = 0.026 for recurrence, P = 0.028 for survival). While in the group of HCC with tumor size>5cm, T2DM showed no significant difference (P = 0.094 for recurrence, P = 0.125 for survival). The mechanism underlying the impact of DM on HCC prognosis was still unclear. Insulin resistance and hyperinsulinemia could result in increasing of IGF-1. Both insulin and IGF-1 promoted proliferation and inhibited apoptosis by receptor-mediated pathways, which may lead to carcinogenesis [39].

This study focused on the impact of p-Ser9-GSK-3β on the prognosis of patients with HCC and the interaction among the expression level of p-Ser9-GSK-3β, T2DM and the prognosis of HCC. Further studies are needed to confirm the interconnection or explain the complex mechanisms among them. We have began a new study aimed at finding this potential mechanism by detecting the expression levels of other related molecules in multiple pathways involved in IGF, NF-kB, PI 3-kinase/Akt and Wnt/β-catenin pathways [40], [41].

In conclusion, we firstly reported p-Ser9-GSK-3β was an independent prognostic factor of patients with HCC and it might mediate the influence of type 2 diabetes mellitus on the prognosis of patients with HCC.

## Supporting Information

Figure S1 **Kaplan-Meier analysis of TTR and OS stratified by T2DM.** (A,C) Kaplan-Meier curves of p-Ser9-GSK-3β expression level in the subgroup of without T2DM, (A) overall survival or (C) recurrence of HCC patients; (B,D) Kaplan-Meier curves of p-Ser9-GSK-3β expression level in the subgroup of with T2DM, (B) overall survival or (D) recurrence of HCC patients.
(JPG)

Table S1 **Univariate analysis for overall survival.**
(DOCX)
