# Supplementary figures and images for: Glycogen Synthase Kinase-3β Is Associated with the Prognosis of Hepatocellular Carcinoma and May Mediate the Influence of Type 2 Diabetes Mellitus on Hepatocellular Carcinoma
Source: PLoS One. 2014 Aug 26;9(8):e105624. doi: 10.1371/journal.pone.0105624 (PMC4144855; doi:10.1371/journal.pone.0105624)

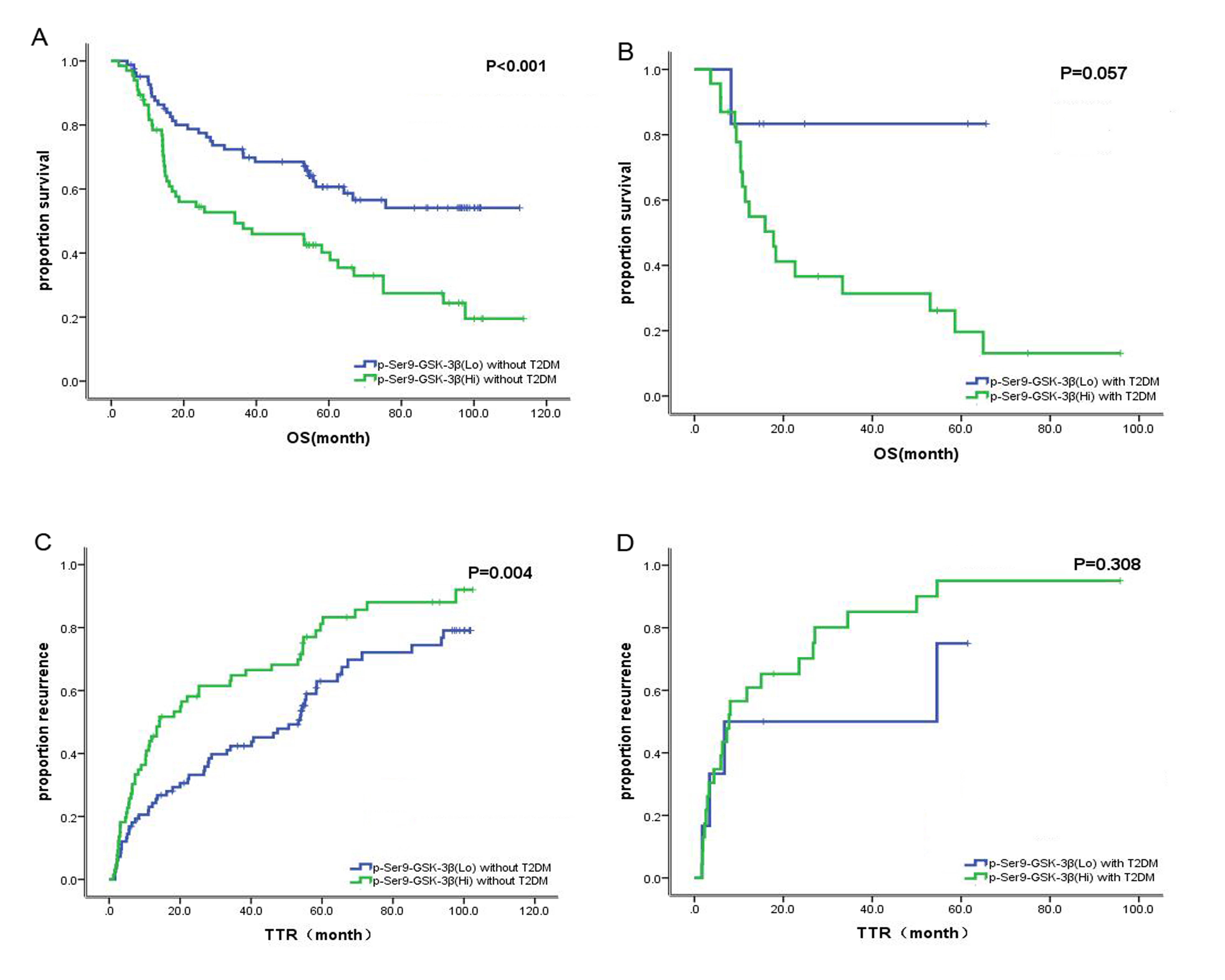

Supplement: Figure S1 — Kaplan-Meier analysis of TTR and OS stratified by T2DM. (A,C) Kaplan-Meier curves of p-Ser9-GSK-3β expression level in the subgroup of without T2DM, (A) overall survival or (C) recurrence of HCC patients; (B,D) Kaplan-Meier curves of p-Ser9-GSK-3β expression level in the subgroup of with T2DM, (B) overall survival or (D) recurrence of HCC patients. (JPG) [file pone.0105624.s001.jpg]
