# Supplementary material for: Glycogen Synthase Kinase-3β Is Associated with the Prognosis of Hepatocellular Carcinoma and May Mediate the Influence of Type 2 Diabetes Mellitus on Hepatocellular Carcinoma
Source: PLoS One. 2014 Aug 26;9(8):e105624. doi: 10.1371/journal.pone.0105624 (PMC4144855; doi:10.1371/journal.pone.0105624)
Supplement: Table S1 — Univariate analysis for overall survival. (DOCX) [file pone.0105624.s002.docx]

**Supplemental Table 1. Univariate analysis for overall survival**

| Variable | Univariate | | |
| --- | --- | --- | --- |
|  | HR | 95%CI | P value |
| Age | 0.999 | 0.982-1.016 | 0.915 |
| Gender: Male/Female | 1.513 | 0.881-2.597 | 0.133 |
| HBsAg: Positive/Negative | 0.723 | 0.403-1.298 | 0.278 |
| HBeAg: Positive/Negative | 0.962 | 0.562-1.646 | 0.888 |
| TBL(umol/l) | 1.005 | 0.979-1.032 | 0.700 |
| Alb(g/dl) | 0.994 | 0.962-1.028 | 0.741 |
| ALT(U/L) | 1.002 | 0.996-1.007 | 0.570 |
| WBC | 0.965 | 0.861-1.081 | 0.54 |
| RBC | 0.951 | 0.663-1.364 | 0.785 |
| PLT(*109/L) | 1.001 | 0.998-1.005 | 0.559 |
| INR | 0.931 | 0.763-1.256 | 0.824 |
| CR | 1.006 | 0.992-1.019 | 0.410 |
| MELD score | 0.846 | 0.686-1.042 | 0.116 |
| AFP level: > 400 /<400 ng/ml | 1.688 | 1.093-2.781 | 0.024 |
| Edmondson-Steiner grade: III or IV / I or II | 1.043 | 0.710-1.522 | 0.830 |
| Liver Cirrhosis: Yes/No | 1.605 | 1.124-2.436 | 0.038 |
| Tumor Encapsulation: presence/absence | 0.779 | 0.502-1.211 | 0.268 |
| Tumor Size: > 5 /< 5 cm | 1.846 | 1.235-2.760 | 0.003 |
| p-Ser9-GSK-3β Expression: High/Low | 2.477 | 1.627-3.769 | 0.001 |
| T2DM: Yes/No | 2.016 | 1.226-3.314 | 0.006 |
| Microvascular Invasion: Yes/ No | 1.731 | 1.083-2.657 | 0.011 |
| Tumor Number: Multiple/solitary | 1.674 | 1.167-3.251 | 0.022 |

Abbreviations: AFP, alpha-fetoprotein; HBsAg, hepatitis B surface antigen; HBeAg, Hepatitis E antigen; TBIL total bilirubin; ALB, albumin; ALT, alanine; WBC, white blood cell; RBC, red blood cell; PLT, platelet count; INR, international normalized ratio; CR, creatinine; MELD, model for end-stage liver disease; T2DM, type 2 diabetes mellitus.
